# Intervention Effects on Adolescent Physical Activity in the Multicomponent SPACE Study: A Cluster Randomized Controlled Trial

**DOI:** 10.1371/journal.pone.0099369

**Published:** 2014-06-12

**Authors:** Mette Toftager, Lars B. Christiansen, Annette K. Ersbøll, Peter L. Kristensen, Pernille Due, Jens Troelsen

**Affiliations:** 1 Institute of Sports Science and Clinical Biomechanics, University of Southern Denmark, Odense, Denmark; 2 Centre for Intervention Research in Health Promotion and Disease Prevention, National Institute of Public Health, University of Southern Denmark, Copenhagen, Denmark; Arizona State University, United States of America

## Abstract

**Background:**

Multicomponent school-based interventions have the potential to reduce the age-related decline in adolescents' physical activity (PA), yet there is not consistent evidence to guide non-curricular and school environment interventions. The aim of this study was to assess the effectiveness of a multicomponent environmental school-based intervention, designed to reduce the age-related decline in PA among adolescents.

**Methods:**

A cluster randomized controlled trial was conducted with 7 intervention and 7 control schools. Baseline measurements were carried out in spring 2010 with 2 years of follow-up. A total of 1,348 students (11–13 years, in grade 5 and 6) enrolled in the study at baseline. The 14 schools included in the study were located in the Region of Southern Denmark. The intervention consisted of organizational and physical changes in the school environment with a total of 11 intervention components. The primary outcome measure was overall PA (cpm, counts per minute) and was supported by analyses of time spent in MVPA, and time spent sedentary. Furthermore, a secondary outcome measure was PA in school time and during recess. PA was measured using accelerometer (Actigraph GT3X).

**Results:**

A total of 797 students completed the trial and had valid accelerometer data. No significant difference was found for overall PA with an adjusted difference of −19.1 cpm (95% CI: −93, 53) or for school time activity with an adjusted difference of 6 cpm (95% CI: −73, 85). A sensitivity analysis revealed a positive significant intervention effect of PA in recess with an adjusted difference of 95 cpm.

**Conclusions:**

No evidence was found of the overall effect of a non-curricular multicomponent school-based intervention on PA among Danish adolescents. The intervention was positively associated with PA during school time and recess, however, with small estimates. Lack of effect on overall PA could be due to both program theory and different degrees of implementation.

**Trial Registration:**

www.Controlled-Trials.com ISRCTN79122411

## Introduction

Adolescence is a period of physical activity (PA) decline [1], [2]. As young people spend a large proportion of their waking hours at school, schools have long been recognized as potentially effective settings for public health initiatives including PA interventions [3], [4]. Studies in school-based PA interventions have reported varying results and there is still a need for high quality and systematic studies among adolescents [3], [5]–[7]. The development of multicomponent interventions (interventions with several intervention components), originates from principles of social ecological models to behavior change, which state that as health behavior is influenced at multiple levels (individual, social, environmental and policy levels), so should interventions in order to maximize effectiveness [8]. Several studies have used the social ecological framework for the design of PA interventions [6], [9]–[12]. The multicomponent intervention study *SPACE for physical activity* has likewise been developed based on a social ecological framework [12]. The overall purpose of the SPACE study was to design, develop, document and assess a comprehensive intervention in schools to promote everyday PA among adolescents. The intervention consisted primarily of non-curricular and environmental components.

The purpose of the current study was to assess the effectiveness of the intervention in reducing the decline in PA among students in the period from grade 5 and 6 (11–13 years) to grade 7 and 8 (13–15 years). The effect of the intervention was estimated with regards to overall PA as well as moderate and vigorous PA (MVPA) and sedentary time. Furthermore, the effect on PA in school time and during recess was investigated.

## Methods

### Study design, setting and participants

The SPACE study used a cluster randomized controlled study design. In 2009, all municipalities in the Region of Southern Denmark were invited to participate in the SPACE study. Five municipalities out of 22 accepted the invitation and were asked to enroll public schools that contained grade 8. A total of 28 schools were recommended or deemed eligible by the municipalities. Among the proposed schools, the project group excluded schools if: a) they were placed in the countryside and had more than 50% of all students living further than 2 km Euclidian distance from the school, and b) the majority of all students were non-native Danish. The research team excluded five schools based on the above-mentioned criteria. This resulted in the enrolment of 23 schools in the five municipalities. The audit tool consisted of a total of eight school characteristics comprising four objective and four subjective characteristics of the 23 schools. The objective characteristics were: 1) Euclidian distance from residence to school for grades 5 and 6, 2) area household income, 3) area education level, and 4) area ethnicity distribution. The information was obtained from Statistics Denmark and by use of a Geographic Information System (GIS). The subjective information, which was based on interviews with municipality consultants and managing school personnel at each school site, consisted of the following variables: 1) school district urbanicity, 2) condition and characteristics of school outdoor areas, 3) school health policy, and 4) level of active transport in the local area. After meetings with schools and visits to the school sites, two schools declined to participate in the project, resulting in 21 eligible schools. A Spearman rank correlation analysis was conducted of the standardized values of the eight variables between the 21 schools to identify the best matches. Based on this 14 schools were matched into 7 pairs and randomized to an intervention or a control group.

Due to the limited number of schools, a matched pair design was applied to optimize the randomization. In order to increase power an unmatched analysis of the matched data was adopted. Diehr et al [13] recommend that for 3–9 pairs, either to use an unmatched design and unmatched analysis or a matched design and unmatched analysis. Matching is helpful in balancing important characteristics between intervention and control groups [13].

**Figure 1:**
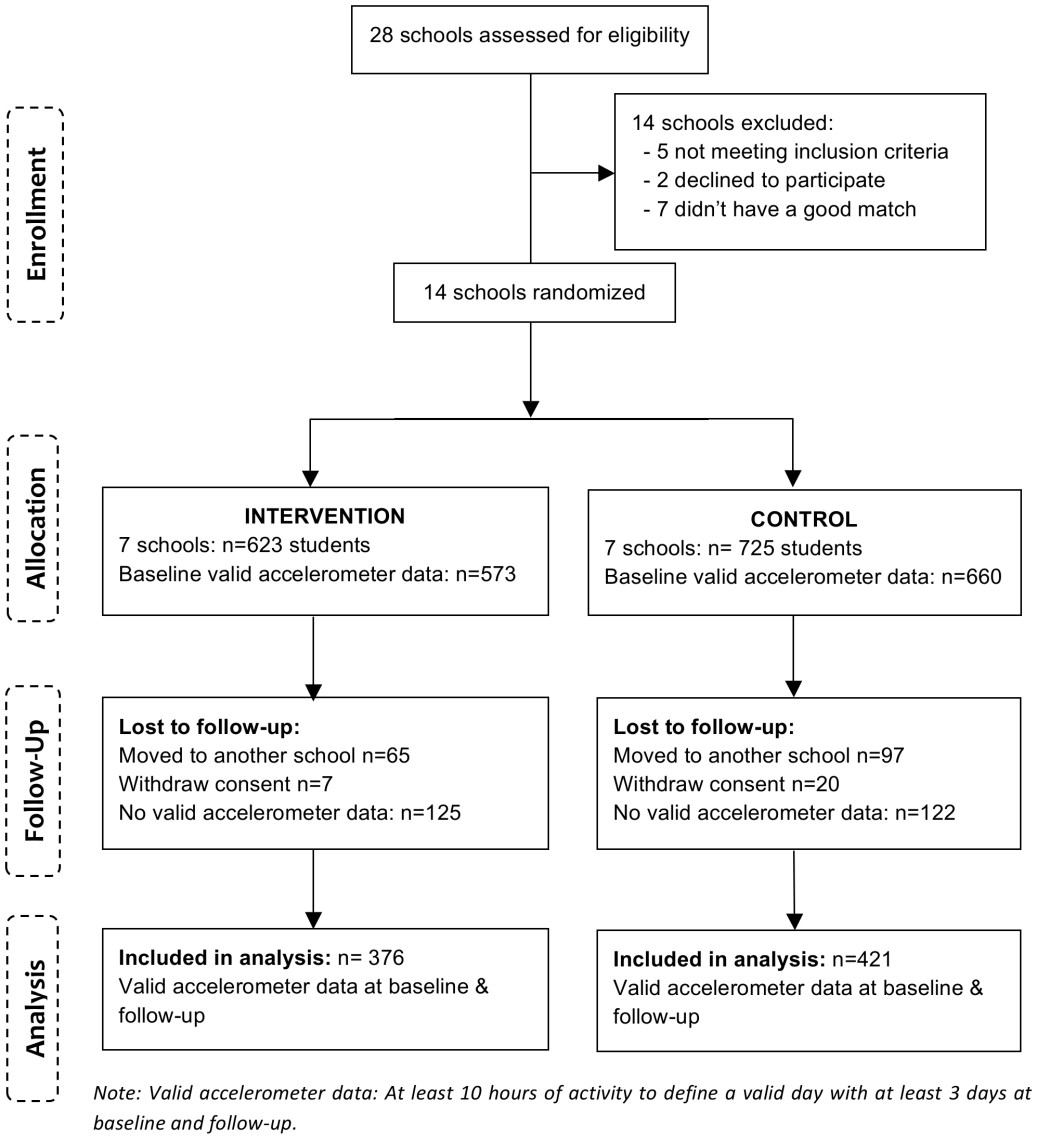
Flow diagram of participants.

A total of 1,348 adolescents in grade 5 and 6 (11–13 years old, mean age 12.5 years, 48.4% girls) entered the study (Figure 1). Further information on the enrolment procedure and the study design has been described in detail elsewhere [14].

### Estimation of sample size

Sample size calculations were performed prior to the study. Conventional levels of statistical power (0.8), level of significance (0.05) and a two-sided test were used. The minimum detectable effect size between groups was determined at 60 counts per minute, representing an approximate 10% difference in the outcome measure at follow-up. We used data from the Danish part of the European Youth Heart Study (EYHS) [15] to estimate the variability in the change in physical activity from baseline to follow-up. Data from the EYHS was also used to estimate the between school variation in physical activity in order to take into account the clustering within schools (ICC = 0.011). The between school variation was estimated while controlling for most of the school matching variables described above. All analyses were performed with statistical software STATA v10 using the modules Sampsi and Sampclus. The calculations showed that a minimum of 12 schools (6 control and 6 intervention schools) were required, based on an average number of 100 students per school.

### Ethical approval

The Danish National Committee on Health Research Ethics reviewed the study protocol and concluded that formal ethical approval was not required. The study was registered and listed in the Danish Data Protection Agency (reference number: 2009-41-3628) and registered in The Current Controlled Trials (ISRCTN79122411, http://www.controlled-trials.com/ISRCTN79122411). Personalized written information about the SPACE study was distributed to parents and students. Parents of the participating adolescents received a passive informed consent form that explained the nature and procedures of the study. Both adolescents and parents were informed that it was possible to withdraw at any stage of the study. This procedure has been found to be ethically appropriate in low-risk research in adolescents [16].

### Intervention

The intervention consisted of 11 intervention components changing the physical and organizational environment of the schools. The multicomponent intervention was developed according to social ecological models of behavioural change [8], [10] and constructed in accordance with existing knowledge-based research and practical experiences from Danish school settings [17]. A detailed written description of the intervention components was delivered to all participating schools and included four physical environment changes and seven organizational environment changes as described in Table 1. The physical environment changes required included the following components: 1) upgrade existing outdoor areas at the school for PA, including unfixed equipment, 2) develop and build playgrounds specially designed for adolescents: play spots, 3) improve safety for active transport to and from school, 4) establish an after school fitness program. The organizational environment changes included: 5) formulate and implement school PA policy, 6) educate teachers as “kick-starters”, who facilitate and motivate PA during recess, 7) establish school play patrol: older students were trained to initiate play and games for minors during school recess, 8) mandatory outdoor recess and/or free access to gym/sports hall, 9) school traffic patrol: older students helped minors cross the streets near the school, 10) educate and train students in safe cycling, and finally 11) school project/theme week once a year focusing on learning about and doing PA during school lessons. Further details of the intervention components are described in the study design protocol [14] and at the project website [18].

**Table 1:** Intervention components and implementation.

Intervention components		Implementation in intervention schools						
		1i	2i	3i	4i	5i	6i	7i
*Physical environment changes*								
1	Upgrade existing outdoor areas at the school for PA including unfixed equipment	+	+	+	+	+	+	+
2	Develop and build specially designed playgrounds for adolescents: Play spots	+	+	+	+	+	+	+
3	Improve safety for active transport to/from school			(+)		+	+	
4	Establish an after school fitness program			+		+		
*Organizational environment changes*								
5	Formulate and implement school PA policy	+	+	+	+	+	+	+
6	Educate teachers as “kick-starters”, who facilitate and motivate PA during recess	+	+	+	+	+	+	+
7	Implement school's play patrol: older students trained to initiate play and games for minors during school's recess	+	+	(+)	+	+	+	(+)
8	Establish mandatory outdoor recess and/or free access to school gym/sports hall during recess	+	+	+	+	+	+	+
9	Establish school's traffic patrol: older students help minors cross the streets near the school		(+)	(+)	(+)		(+)	
10	Educate and train students in safe cycling	(+)	(+)	(+)	(+)	(+)	(+)	(+)
11	Implement school project/theme week once a year focus on learning about and doing PA during school lessons	+	+	+	+	+	+	+

+ *Implemented in intervention period, (+) Implemented before intervention start.*

### Implementation

The implementation of the intervention began in autumn 2010. All intervention schools upgraded the outdoor areas (costing €10,000–20,000) and established play spots (costing €65,000–250,000). Furthermore, as documented in the process evaluation [17], PA policy, kick-starters, mandatory outdoor recess/open sports hall, and school theme week were implemented in all intervention schools. The school play patrol was already implemented at two schools prior to involvement in the intervention. School traffic patrol was already implemented in four of the schools. In the remaining three schools it was not relevant because traffic authorities have ranked the access to the schools as safe. Cyclist education was already implemented at all seven schools, and did not directly change, apart from being included in the school's PA policy. Improvement of cycling infrastructure was partly met at two schools, but lack of financial support made it impossible to implement this component in the remaining five schools. The organization of the after school fitness program was implemented in two local school areas. Lack of voluntary instructors made the component impossible to implement in the other five areas (Table 1).

### Data collection

Baseline measurements were obtained in spring (April to June) 2010 among all students in grade 5 and 6 in seven intervention and seven control schools, with follow-up measurements in spring 2012. PA was objectively assessed among all students using accelerometers (Actigraph GT3X). Sex and age were obtained through school records. The parental income was obtained through Statistics Denmark using the Danish Civil Registration System, which monitors individual level information such as address and income [19], [20]. The income was dichotomized as above or below the relative poverty line using 50% of median household income as the threshold [21]. Similarly, parents' ethnicity (both born in Denmark) was obtained through the Danish Civil Registration System. The adolescents' height and weight were objectively measured by the research team using standard anthropometric procedures. Overweight was defined using sex and age specific BMI cut points relative to 25 kg/m^2^ for adults [22].

### Accelerometer data reduction

The adolescents were instructed to wear the accelerometers all waking hours for seven consecutive days except when doing water activities. The accelerometers were downloaded using Actilife (Actigraph) and analyzed by the software program Propero (University of Southern Denmark). Data were analyzed using 30 seconds of epoch, and activity for all 24 hours was included. Strings of 60 minutes or more of consecutive zeroes, allowing for two epoch periods of non-zero interruptions, were interpreted to represent non-wear time and were excluded from each individual recording [23]. Adolescents with valid accelerometer data i.e. at least 3 days with at least 10 hours (600 min) of activity recorded per day, at both baseline and follow-up were included in this study (n = 797). Data were aggregated at the individual level as mean measures on weekdays and weekend days. The average number of minutes per day that the participants wore the accelerometer and the number of activity counts per minute (cpm) were calculated. To investigate time in different PA intensities the Evenson activity cut points were used [24]. These cut points have been recommended and validated in previous studies [25]. Sedentary time (≤100 cpm) and MVPA (≥2296 cpm) were expressed as minutes per day of accelerometer activity. Information on schools' timetables and periods of recess with exact bell times was obtained from each of the participating schools and merged with the accelerometer data.

### Statistical analyses

Lost to follow-up analyses were performed for baseline outcome measures and background characteristics using logistic (binary outcome such as sex) and linear (continuous outcome such as age) regression models for each of the background variables (sex, age, BMI, weight status, household income and parents ethnicity) and for each of the outcome measures on PA and sedentary time. Lost-to-follow status was included as exposure. Clustering of students within schools was accounted for by including schools as a random effect in the analyses.

**Figure 2:**
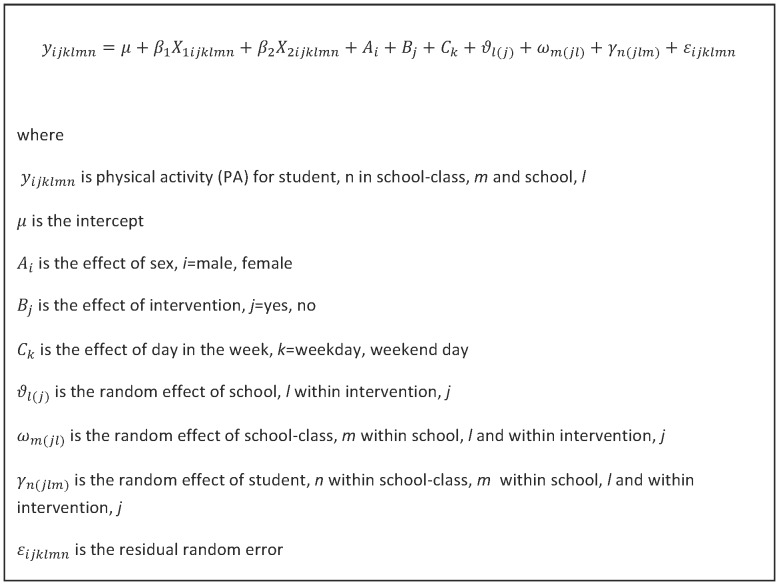
Regression model for the primary outcome, physical activity (cpm).

When estimating effects multilevel linear mixed models were used taking into account clustering of students within classes and classes within schools. The primary outcome measure was overall PA (cpm) and was supported by analyses of time spent in MVPA, and time spent sedentary. Furthermore, a secondary outcome measure was PA in school time and during recess. The outcome measure at follow-up was used as the dependent variable and the intervention condition was the explanatory variable. Analyses were adjusted for differences in baseline PA, sex and age at baseline. The primary outcome measures were derived for weekdays and weekend days, whereas the secondary outcome measures were only derived for weekdays. Students were measured for seven consecutive days, five weekdays and two weekend days. In the analyses of overall PA a random effect for student was included with an aggregated measure for weekdays and an aggregated measure for weekends. Therefore, for each student we had two observations. The random effect of school and classes within schools were included in the primary and secondary analyses. Furthermore, the random effect of students within classes and schools was included in the primary analyses. The primary analyses were also adjusted for differences in weekday/weekend day. Figure 2 shows the regression model for the primary outcome. We used the intention to treat principle in that it analyses the individuals in the groups to which they were originally assigned [26].

Effect modification was explored for sex, parental income, weight and PA at baseline by adding one interaction term at a time in the multilevel model. Residual plots were conducted to evaluate the model assumptions (i.e. normal distribution of residuals and equal variances). When model assumptions were not met outcome variables were analyzed using a log-transformation. When no differences in the effect of intervention and p-values were seen, results were reported without the log-transformation. When log-transformation of the outcome was necessary (due to lack of fulfilled model assumptions), the intervention effect was estimated as the difference between the back-transformed least square mean estimates of log(PA) for the intervention and control groups (all other effects in the model than intervention were kept at the mean value). Due to the use of log-transformation, confidence intervals cannot be estimated for the difference in PA between the intervention and control groups [27].

To interpret the variation between schools, classes within schools and students within classes and schools, intraclass correlation coefficients (ICC) were calculated.

Sensitivity analyses were conducted with at least one valid accelerometer day as the inclusion criteria. There were only minor differences in the results, and results based on the three days inclusion criterion are reported.

**Figure 3:**
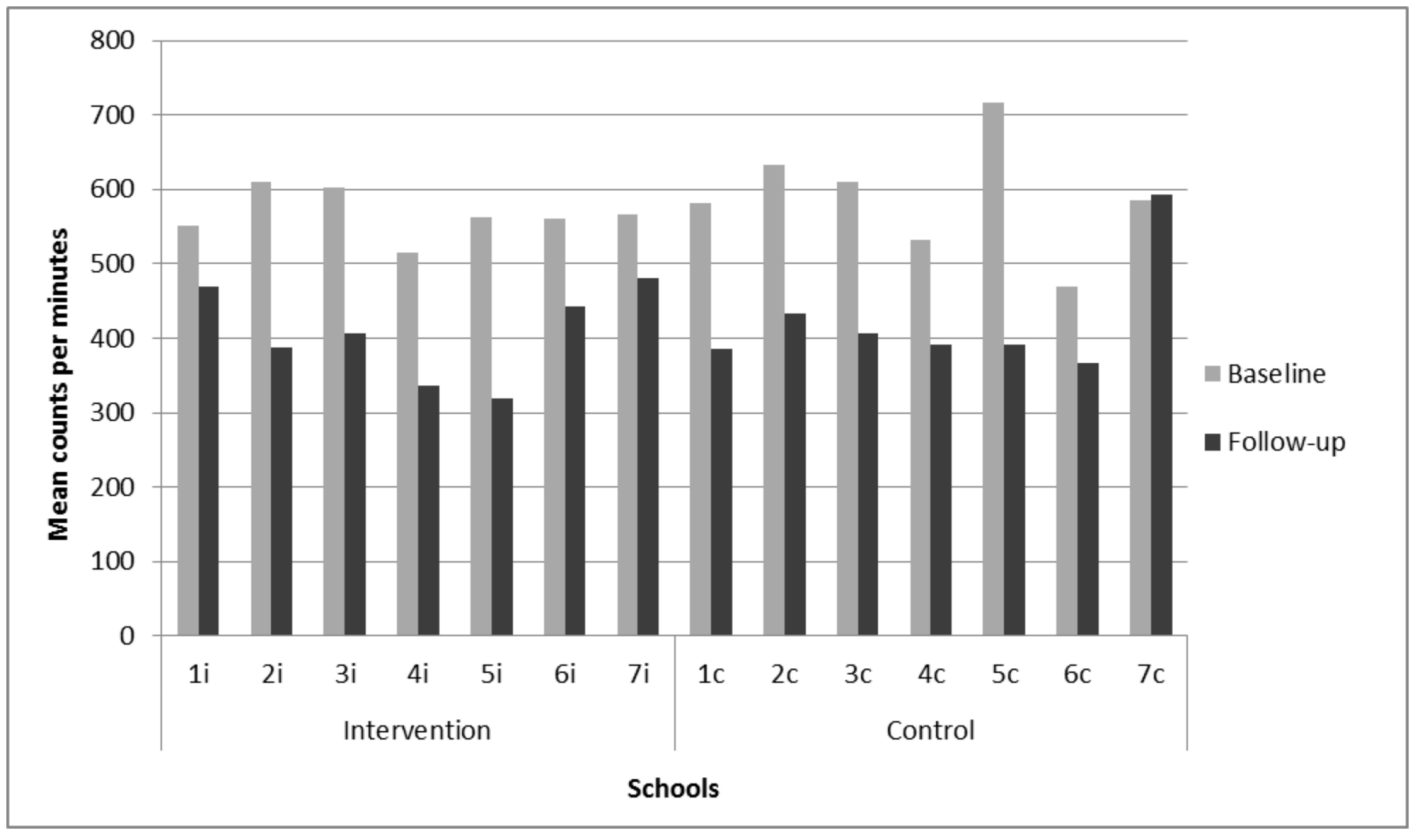
Physical activity (cpm) in the intervention (n = 376) and control (n = 421) schools at baseline and follow-up.

Finally, descriptive analyses of PA (cpm) at each school were reported to illustrate the variance between schools. One school (school c7 in Figure 3) had a markedly higher PA level at follow-up compared to the other schools. Information from the students and the school staff showed that many students from this school had attended an outdoor local music festival in the same week of PA measurement, which was a plausible explanation for the very high level of activity. Based on this it was decided to conduct a sensitivity analysis, excluding the specific school from the analysis.

Data were analyzed by Stata/SE, version 12. A 5% significance level was used.

The protocol for this trial and supporting CONSORT checklist are available as supporting information; see Checklist S1 and Protocol S1.

## Results

### Participants

A total of 1,348 students entered the study of which 1,233 (91%) provided valid accelerometer data at baseline (Figure 1). Out of the participating adolescents at baseline 13% (n = 162) had moved to another school at follow-up, and 2% (n = 27) withdrew consent. A total of 797 adolescents (65%) had at least three days of valid accelerometer data at baseline and follow up and were included in the follow-up analyses. Average daily accelerometer wear time was 14 hours and number of valid days was 6.6 in both the intervention and control group. Average BMI was 18.7 kg/m^2^ and the proportion of overweight was 11.7% and 13.8% in intervention and control group, respectively. Most parents (>90%) were born in Denmark and 13% had a low household income (Table 2). There were on average 4.8 classes per school with a minimum of 4 and a maximum of 6 classes. There were on average 11.2 students in each class with valid data at both baseline and follow-up with a minimum of 3 and a maximum of 20 students (data not shown).

**Table 2:** Baseline characteristics of participants.

	Intervention (n = 376)	Control (n = 421)
Girls, %	51.3	47.9
Age, years	12.5 (0.63)	12.5 (0.61)
BMI, kg/m^2^	18.7 (2.6)	18.6 (3.0)
Overweight^a^, %	11.7	13.8
Low household income^b^, %	13.0	13.3
Parents' native Danish^c^, %	91.2	93.1
Daily accelerometer wear time, hours	14.2 (1.10)	14.0 (1.02)
Number of valid accelerometer days	6.7 (1.25)	6.6 (1.11)

M(SD) unless otherwise stated. N = 797

a *Sex and age standardized cut points* [*22*] *. ^b^Household income lower than 50% of the sample median income. ^c^Both parents native Danish. n varies due to different data sources*

### Lost to follow-up

The differences between students included in analyses and students lost to follow-up are shown in Table 3. The lost to follow-up group was defined as students who moved to another school, withdrew their consent or had missing data at baseline or follow-up accelerometer measurements (Figure 1). The lost to follow-up group consisted of fewer girls (42.1% vs. 52.8%), was a little older (12.7 years vs. 12.5 years), was more likely to have a higher BMI (19.2 vs. 18.8), and more likely to be from low economic background (23.0% vs. 14.1%) at baseline compared to adolescents included in the analysis. Regarding the outcome measures, the lost to follow-up group had a higher level of PA in school time (581 vs 547cpm) (Table 3). No significant differences were observed between the intervention and control group among individuals lost to follow-up (data not shown).

**Table 3:** Baseline characteristics individuals lost to follow-up and with complete data.

	Lost to follow-up or missing data (n = 551)	Complete data (n = 797)	p-value
Girls, %	42.1 (38.0, 46.3)	52.8 (49.4, 56.3)	<0.001
Age, years	12.7 (12.6, 12.8)	12.5 (12.5, 12.6)	<0.001
BMI, kg/m^2^	19.2 (18.8, 19.6)	18.8 (18.4, 19.1)	0.008
Overweight^a^, %	16.7 (12.6, 20.7)	13.8 (10.2, 17.5)	0.160
Low household income^b^, %	23.0 (18.7, 27.3)	14.1 (10.2, 18.0)	<0.001
Parents' native Danish^c^, %	89.9 (84.9, 94.9)	90.5 (85.7, 95,3)	0.692
Overall PA, cpm	574.7 (543.2, 606.2)	582.1 (553.5, 610.7)	0.595
Time in MVPA/day, min	60.1 (56.7, 63.4)	57.5 (54.5, 60.5)	0.087
Time in sedentary activity/day, min	511.7 (504.6, 518.7)	509.0 (503.7, 514.6)	0.577
School time PA, cpm	581.1 (532.9, 629.2)	547.5 (500.6, 594.4)	0.003
PA in recess, cpm	1032.6 (901.3, 1163.9)	976.5 (848.8, 1104.3)	0.078

**M (CI 95%) unless otherwise stated.**

*Based on a regression model adjusted for cluster on school level.*

*PA: physical activity. MVPA: moderate to vigorous physical activity. Cpm: counts per minute*

a *Sex and age standardized cut points* [*22*] *. ^b^Household income lower than 50% of the sample median income. ^c^Both parents native Danish*

### Physical activity level

Table 4 shows PA in the intervention and control group at baseline and follow-up and the adjusted intervention effect. The 2-year follow-up time resulted in an average decrease in PA for all PA outcome measures. For overall PA, the crude decrease during the 2-year period was 157 cpm in the intervention group and 162 cpm in the control group. Time spent in MVPA was 57 minutes/day at baseline and declined to 44 minutes and 48 minutes in the intervention and control group, respectively. Sedentary time increased in both groups with 1 hour (daily accelerometer wear time remained the same at baseline and follow-up, approx. 14 hours). Adjusted for baseline values of sex, age, weekend/weekdays and clustering, the difference between intervention and control was −19 cpm of overall PA (95% CI: −93, 53), -3 minutes/day in MVPA (95% CI: −15, 8), and for sedentary time the adjusted effect was zero (95% CI: −20, 22).

**Table 4:** PA in the intervention (n = 376) and control (n = 421) group at baseline and follow-up.

	Baseline	Follow-up	Crude difference within groups	Adjusted difference between groups	p-value	ICC
	*M (SD)*	*M (SD)*		*(95% CI)*		
**Overall PA, cpm**						
*Intervention*	566 (221)	408 (167)	−158	−19.1 (−92.6, 53.1)^a^	0.591	0.09 (school)
*Control*	596 (252)	433 (206)	−163			0.11 (class)
						0.42 (student)
**MVPA time, min/day**						
*Intervention*	57 (24)	44 (23)	−13	−3.3 (−15.4, 8.7)^a^	0.587	0.15 (school)
*Control*	57 (24)	48 (27)	−9			0.17 (class)
						0.32 (student)
**Sedentary time, min/day**						
*Intervention*	515 (81)	575 (88)	60	0.1 (−20.0, 21.7)^a^	0.938	0.02 (school)
*Control*	503 (100)	565 (106)	62			0.04 (class)
						0.35 (student)
**Weekday PA, cpm**						
*Intervention*	574 (222)	422 (173)	−152	−8.4 (−67.2, 50.3)^b^	0.782	0.08 (school)
*Control*	601 (250)	439 (203)	−162			0.13 (class)
**Weekend PA, cpm**						
*Intervention*	555 (357)	345 (225)	−210	−40.3 (−141.3, 60.7)^b^	0.433	0.11 (school)
*Control*	596 (406)	417 (313)	−179			0.12 (class)
**School time PA, cpm**						
*Intervention*	554 (221)	413 (187)	−141	5.8 (−73.1 84.7)^b^	0.886	0.15 (school)
*Control*	533 (196)	395 (202)	−138			0.29 (class)
**Recess PA, cpm**						
*Intervention*	1005 (546)	662 (402)	−342	19.2 (−156.3,194.7)^b^	0.834	0.11 (school)
*Control*	929 (650)	617 (507)	−311			0.27 (class)

a *Effect analyses adjusted for baseline PA, sex, age, weekend/weekdays. School, class and student were included as random effects to account for clustering. ^b^Effect analyses adjusted for baseline PA, sex, age. School and class included as random effects to account for clustering. PA: physical activity, MVPA: moderate and vigorous physical activity. Cpm: counts per minute, ICC: Intraclass correlation*

Stratifying the analyses by weekdays and weekend days showed lower PA at weekends compared to weekdays, especially at follow-up. PA in school time declined with 141 cpm in intervention group and 138 cpm in the control group, and no significant intervention effect was found (6 cpm, 95% CI: −73, 85). Recess PA for the intervention group declined from an average of 1005 cpm at baseline to 662 cpm at follow-up, and the control group PA declined from 929 cpm to 617 cpm, and no significant intervention effect was found (19 cpm, 95% CI: −156, 195) (Table 4). No significant interactions were seen between intervention and sex, parental income, weight and PA at baseline.

The intervention effect was further explored by analyses stratified by sex, household income, overweight and PA level at baseline. No effect of the intervention was seen in these analyses. In general, boys were more active than girls, and overweight adolescents less active than normal weight adolescents. The intervention did not change this pattern from baseline to follow-up (data not shown).

Variation in PA between schools was explored and revealed considerable differences across schools during the 2-year study period (Figure 3). The biggest difference was seen in school 5i and 5c, with a 45% decline in average PA level (cpm). The smallest decline (15%) was observed for the two intervention schools, 1i and 7i. The control school 7c increased PA from baseline to follow-up most likely due to a local music festival (Figure 3). A sensitivity analysis investigating the intervention effect, when excluding school 7c showed positive intervention effects for all outcomes (non-significant for most parts). For overall PA, the effect was 12 cpm (95% CI: −35, 60) and for school time PA the effect was 29 cpm (95% CI: −43, 101). A significant effect for PA in recess was observed (p = 0.046) with an estimated difference at 95 cpm (Table 5).

**Table 5:** Sensitivity analysis, school c7 excluded.

	Baseline	Follow-up	Crude difference within groups	Adjusted difference between groups	p-value	ICC
	*M (SD)*	*M (SD)*		*(95% CI)*		
**Overall PA, cpm**						
*Intervention*	566 (221)	408 (167)	−158	12.3 (−34.9, 59.5)^a^	0.611	0.03 (school)
*Control*	598 (247)	400 (191)	−198			0.05 (class)
						0.42 (student)
**MVPA time, min/day**						
*Intervention*	57 (24)	44 (23)	−13	2.2 (−4.2, 8.6)^a^	0.497	0.04 (school)
*Control*	57 (24)	42 (21)	−15			0.07 (class)
						0.26 (student)
**Sedentary time, min/day**						
*Intervention*	515 (81)	575 (88)	60	−6.0 (−20.3, 8.3)^a^	0.413	0.01 (school)
*Control*	501 (70)	584 (77)	83			0.02 (class)
						0.31 (student)
**Weekday PA, cpm**						
*Intervention*	574 (222)	422 (173)	−152	16.8 (−24.0, 57.6)^b^	0.421	0.02 (school)
*Control*	601 (235)	410 (193)	−191			0.07 (class)
**Weekend PA, cpm**						
*Intervention*	555 (357)	345 (225)	−210	2.0 (−62.6, 66.7)^b^	0.952	0.03 (school)
*Control*	606 (424)	366 (290)	−240			0.03 (class)
**School time PA, cpm**						
*Intervention*	554 (221)	413 (187)	−141	28.6 (−43.4, 100.7)^b^	0.436	0.11 (school)
*Control*	552 (200)	370 (189)	−182			0.27 (class)
**Recess PA, cpm**						
*Intervention*	1005 (546)	662 (402)	−343	95.0*^b^	0.046	0.04 (school)
*Control*	967 (550)	584 (524)	−383			0.20 (class)

**PA in the intervention (n = 376) and control (n = 344) group at baseline and follow-up.**

a Effect analyses adjusted for baseline PA, sex, age, weekend/weekdays. School, class and student were included as random effects to account for clustering. ^b^Effect analyses adjusted for baseline PA, sex, age. School and class included as random effects to account for clustering.^*^Back-transformed estimate based on analysis of log-transformed outcome. Due to log-transformation, no confidence interval is calculated. PA: physical activity, MVPA: moderate and vigorous physical activity. Cpm: counts per minute, ICC: Intraclass correlation.

## Discussion

The aim of this study was to investigate whether an environmental non-curricular intervention in the school setting was able to change the level of PA in Danish adolescents. The overall effect evaluation showed no significant intervention effect. Lack of effect could be due to the design of the intervention, lack of effect in single intervention components, and the implementation of the intervention components. The intervention package in the SPACE study consisted of 11 intervention components, combining physical environment changes with supportive organizational changes. The intervention could be categorized as a complex intervention, with regards to both the number of intervention components and the diverse contents and processes of developing and implementing each of the components. The implementation of several components turned out to be a challenging task for some schools, mainly due to economic reasons. The most successfully implemented intervention components in the SPACE study aimed at increasing PA in recess (e.g. kick-starters, mandatory outdoor recess/open sports hall, upgrading school outdoor areas and establishing Play spots). This seems to be reflected by the positive (but non-significant) intervention effect estimates for PA in school time and recess in the complete case analysis. In the sensitivity analysis, a significant and relative high estimate of 95 cpm was obtained for PA in recess.

The intervention components in the SPACE study were primarily non-curricular but had focus on PA in several arenas. It could be argued that interventions with a more mandatory structure might be more effective on improving the frequency, intensity and duration of PA, e.g. physical education and other curricular interventions as shown in other studies. For example, Kriemler et al concluded that a Swiss school-based multicomponent intervention with compulsory elements improved MVPA in school with 13 min/day and total MVPA with 11 min/day [28].

There seems to be general consensus in recent published reviews and studies on school-based interventions that multicomponent approaches are the most effective way of increasing PA levels in adolescents [3], [5]. In the SPACE study, no overall effect was achieved despite the fact that the study included a large number of components, raising the question that maybe the intervention had too many components and was too complex to implement. This could however also be more related to the content of the intervention components, and not because of the multi-stringed intervention package itself. A possible explanation for the no overall effect could be that the content of some of the intervention components in practice turned out to be more attractive for younger students (grade 3–5) instead of older students (grade 7–8) as intended. This was indicated in qualitative research on the SPACE study, which also showed that it is a challenge to activate adolescents in this age group. Especially the girls perceived several barriers for being physically active, primarily related to their attitude to childish behavior and being sweaty. The analyses of PA in recess also showed that boys in general were more active than girls, which indicate that boys and girls may experience recess in different ways. Boys view recess as an opportunity to play competitive games, while girls may view recess as an opportunity to socialize with friends [17]. This pattern has been found in other studies too [29].

Another possible explanation for not finding an effect could be that PA behavior, like healthy eating habits, are established earlier in childhood and are relatively resistant to changes. Recent successful multicomponent RCT intervention studies that have used accelerometry, have in general focused on younger age groups than in the SPACE study, e.g. the Swiss KISS study [28] and the Norwegian HEIA study [30]. Thus, it can be argued that interventions to increase PA should begin before adolescence [31]. Furthermore, it could be suggested that involvement of the family/parents should play a more significant role of the intervention, as shown effective in other studies [32]. Social network and peer influence in adolescents play an important role for health behavior. Several of the intervention components were developed with a specific focus on the aspect of social relations. The play spots were designed to encourage social interaction among the adolescents as a local “hangout spot”, also after school hours, and the kick-starters initiated team play and activities in recess. The play patrol highlighted the importance of role models, with older students initiating play and games for minors during recess. Nonetheless, it is possible that the intervention could have succeeded with more focus on the social relational environment.

The non-effect for overall PA found in this study is in line with findings from a recent meta-analysis of studies that examined 30 interventions aimed at promoting PA in children. The results indicate a small to negligible pooled effect on total time spent in MVPA (4 minutes a day). The meta-analysis differs from previous systematic reviews with inclusion of only accelerometry as objective measurements of PA [7].

At baseline a high participation rate was obtained with 91% (n = 1,233) of eligible students contributing with valid accelerometer data for at least three days. At follow-up the participation rate dropped to 65% (n = 797) of those participating at baseline. In large-scale school-based interventions a certain dropout rate is expected. Efforts were done to increase participation rate at both baseline and follow-up, e.g. passive informed consent procedure was used and text messages to remind of wearing the accelerometer were sent to students/parents if they signed up for it. To explore if the definitions of lost to follow-up affected the results, the analyses were repeated excluding students who moved to another school (n = 162), and for participants with at least one valid day (n = 965). This did not change the results significantly.

To investigate if the accelerometer data reduction criterion, of at least three valid days had an impact on the effect results, a sensitivity analysis was conducted including all participants with at least one valid day of accelerometer data. This did not change the results of the study, but it did improve the participation rate considerably to 76% (n = 965). Using at least one valid accelerometer day as inclusion criterion thus increased the sample size and statistical power, however, very few studies are reported based on only one valid day. Therefore, to compare with other studies and to increase the validity of the study, the three valid day criterion was used [33].

### Strengths and limitations

A strong aspect of this study is the cluster randomized controlled design and the large sample size. PA was measured objectively and both overall PA and school time PA was assessed. Furthermore, the 2-year follow-up period is considered long, compared to most other intervention studies reported [3], [5].

Limitations of the study relate to the fact that the intervention was implemented with varying degree in the intervention schools. On the other hand, it could be stated that the results from this study reflect the real-life school setting.

The students lost to follow-up were significantly different from those included on several variables. In general, the lost to follow-up group consisted of fewer girls, was a little older, more likely to be from low socioeconomic background and was more likely to have a high BMI at baseline compared to adolescents included in analyses. Thus, we are unable to rule out that the lost to follow-up group may well have benefited from the intervention.

The majority of students at the schools involved were native Danes and all lived in the same region of Southern Denmark. Although the schools and students included in the study may not be representative of the Danish population of schools and students, the lack of association might be valid for the Danish students in general.

The SPACE study fulfils the intention to treat principles in that it analyses the individuals in the groups to which they were originally assigned. The most important reason for conducting intention to treat analyses is that it preserves the randomization, and thus is a defense against bias [26]. For example, there were ten students who moved to another SPACE school in the period from baseline to follow-up. They were all categorized as belonging to the group they were randomized to initially. Furthermore, the intervention effect was analyzed with no regards to implementation status, and not as a per protocol analysis where a trial is evaluated with regards to adherence to the study protocol [34], [35].

Despite the benefits of using accelerometers to assess PA, some limitations should also be addressed, such as the problem of adequately measuring cycling activity. In Denmark, cycling is very common as part of daily active transport. Therefore, this aspect of the SPACE study was evaluated using self-reported measures. Another issue is that accelerometers may be impractical to wear during contact sport. Participants can forget to put the sensor on or choose not to wear it because of concerns related to appearance. Furthermore, shortcomings are that accelerometers do not capture swimming, weight bearing activities (e.g. weight training or stair climbing) and there is a risk of ‘leveling-off’ when running at a high speed. Because of these issues, accelerometers may underestimate PA in adolescents [36]. Despite these limitations, there are still several good reasons for using accelerometers in intervention research compared to self-reported measures. An important aspect is that it is very difficult to measure a specific change in PA with a self-reported measure due to a risk of recall bias and because the details with regards to overall duration, frequency and intensity are difficult to capture in a simple questionnaire [37].

Measurement of PA, as done in the SPACE study, will always be a snapshot of the adolescents' PA behavior, and despite efforts to account for schools to have a “usual” week in the data collection period, external factors, such as a local music festival, can have substantial impact on the results.

### Future studies

Future intervention studies should aim at designing an intervention that builds on the experiences from the SPACE study. Especially the organizational intervention components, for example the kick-starter initiative proved to be a success, according to interview with school staff. The building of play spots and upgrading of school outdoor areas were relatively expensive, and the attempts to improve the physical environment for active transport demanded a high degree of political involvement and support, and such projects take a long time from proposal to implementation, and are therefore not easy to implement nationwide. Finally, and important to mention, the design of the build environment for promotion of PA and active transport in Denmark seems in general rather good compared to many other countries.

Qualitative findings from the process and anthropological part of the SPACE study have been useful in the discussion of the reasons for the lack of effect. The qualitative data collection was conducted after the implementation, and future studies should consider incorporating qualitative work also before the planning and implementation of the intervention in an effort to ensure that the intervention reaches the desired target group and is filling the most important gaps.

Conducting randomized controlled trials in a real life setting is challenging, time consuming and expensive. There are many stakeholders, and many issues should be taken into consideration. Therefore, a solution for researchers could also be focusing on evaluating the effect of natural experiments, for example where planned improvements of the physical environment around schools are accompanied by supportive organizational interventions.

## Conclusions

No evidence was found of the overall effect of a non-curricular multicomponent school-based intervention on PA among Danish adolescents. Lack of effect on overall PA could be due to both program theory and different degrees of implementation. The intervention was positively associated with PA during school time and recess, however with small and non-significant estimates. A sensitivity analysis revealed a significant intervention effect of 95 cpm in recess.

## Supporting Information

Checklist S1 **CONSORT Checklist.**
(DOC)

Protocol S2 **Trial Protocol.**
(PDF)
