# Supplementary material for: Intervention Effects on Adolescent Physical Activity in the Multicomponent SPACE Study: A Cluster Randomized Controlled Trial
Source: PLoS One. 2014 Jun 12;9(6):e99369. doi: 10.1371/journal.pone.0099369 (PMC4055775; doi:10.1371/journal.pone.0099369)
Supplement: Protocol S2 — Trial Protocol. (PDF) [file pone.0099369.s002.pdf]

Toftager et al. BMC Public Health 2011, 11:777
http://www.biomedcentral.com/1471-2458/11/777

BMC Public Health

STUDY PROTOCOL Open Access

SPACE for physical activity - a multicomponent intervention study: study design and baseline findings from a cluster randomized controlled trial

Mette Toftager*, Lars B Christiansen, Peter L Kristensen and Jens Troelsen

Abstract

Background: The aim of the School site, Play Spot, Active transport, Club fitness and Environment (SPACE) Study was to develop, document, and assess a comprehensive intervention in local school districts that promote everyday physical activity (PA) among 11-15-year-old adolescents. The study is based on a social ecological framework, and is designed to implement organizational and structural changes in the physical environment.

Methods/design: The SPACE Study used a cluster randomized controlled study design. Twenty-one eligible schools in the Region of Southern Denmark were matched and randomized in seven pairs according to eight matching variables summarized in an audit tool (crow-fly distance from residence to school for 5-6th graders; area household income; area education level; area ethnicity distribution; school district urbanity; condition and characteristics of school outdoor areas; school health policy; and active transport in the local area). Baseline measurements with accelerometers, questionnaires, diaries, and physical fitness tests were obtained in Spring 2010 in 5-6th grade in 7 intervention and 7 control schools, with follow-up measurements to be taken in Spring 2012 in 7-8th grade. The primary outcome measure is objective average daily physical activity and will be supported by analyses of time spent in moderate to vigorous activity and time spent sedentary. Other secondary outcome measures will be obtained, such as, overweight, physical fitness, active commuting to/from school and physical activity in recess periods.

Discussion: A total of 1348 adolescents in 5-6th grade in the Region of Southern Denmark participated at baseline (n = 14 schools). The response rate was high in all type of measurements (72.6-97.4%). There were no significant differences between intervention and control groups at baseline according to selected background variables and outcome measures: gender ($p = .54$), age ($p = .17$), BMI ($p = .59$), waist circumference ($p = .17$), physical fitness ($p = .93$), and physical activity (accelerometer) ($p = .09$).
The randomization and matched pair design produced equivalent groups according to central outcome measures and background variables. The SPACE for physical activity Study will provide new insights on the effectiveness of multicomponent interventions to improve adolescents' physical activity level.

Trial registration: Current Controlled Trials ISRCTN79122411

Background

The physical, mental, and social health benefits of physical activity (PA) in children and adolescents are well documented[1-4]. Growing evidence suggests that PA in childhood and adolescence will track into adulthood and can prevent lifestyle related diseases such as cardiovascular diseases, some cancers, osteoporosis and diabetes that are manifested later in adulthood[2,4-7]. Despite the benefits of PA, a significant number of young people in Denmark and other Western countries do not reach recommended levels of PA [8,9].

Efforts to increase levels of PA in children and adolescents have primarily relied on community, family or school settings, and individual or educational approaches, but results have been mixed[10-13]. As young people tend to spend a large proportion of their waking hours at

* Correspondence: mto@sdu.dk
Institute of Sports Science and Clinical Biomechanics, University of Southern Denmark, Campusvej 55, 5230 Odense M, Denmark

© 2011 Toftager et al; licensee BioMed Central Ltd. This is an Open Access article distributed under the terms of the Creative Commons Attribution License (http://creativecommons.org/licenses/by/2.0), which permits unrestricted use, distribution, and reproduction in any medium, provided the original work is properly cited.

school, schools have long been recognised as potentially effective settings for public health initiatives, and PA interventions and can largely benefit the most socially disadvantaged groups of children[11,14-16].

In a systematic review from 2007[10] based on 33 intervention studies in children and 24 studies in adolescents(12-18 y), it was concluded that among studies conducted in children, limited and inconclusive evidence for an effect was found. On the other hand, in adolescents, school based interventions involving family, community, and/or multicomponent interventions can increase physical activity in adolescents. However, these conclusions were based on few studies. In the review, out of the 24 studies in adolescents, six of them evaluated a multicomponent intervention, all in the school setting, and only three of these studies were large high quality randomized trials, all showing positive results. Most studies reviewed were conducted in USA, and only a few in a European context. It is concluded that in general there is a need for more systematic and high quality studies[10].

The effect of multicomponent interventions is in keeping with the ecological approach to behaviour change, which states that as health behaviour is influenced at multiple levels, so should interventions to maximize effectiveness. One principle of ecological models is that they need to be tailored to specific behaviours, and has been identified as a suitable conceptual model for the design of PA interventions[10,17-20]. In the past decades, there has been an increasing interest in, and application of, ecological models in research and practice, due in part to the potential in guiding comprehensive approaches to change behaviour [21-23].

The multicomponent intervention study SPACE for physical activity (School site, Play Spot, Active transport, Club fitness and Environment) has been developed based on a social ecological framework. The SPACE study is designed to develop, document and assess a comprehensive intervention in local school districts to promote everyday PA among adolescents. The target group is students in 5-6th grade (11-13 years of age) followed for two years, as several studies show a drastically decrease in the level of PA at the transition to teenage years [24-27]. In the SPACE Study we aim to reduce this age-related decline in PA level.

The research hypothesis to be tested is that a multicomponent PA promotion programme comprising different organizational and physical environmental intervention components can be designed and successfully implemented in collaboration with schools and municipalities in Denmark, and that the intervention will lead to increased PA levels among adolescents.

The purpose of this paper is to present the study design, the design of the intervention components, the data collection procedures, outcome measures and selected baseline characteristics of the SPACE study.

The SPACE study is registered in the Current Controlled Trials (ISRCTN79122411), and findings will be reported in accordance with the guidelines of the CONSORT statement [28].

Methods/Design

Study design

This study employed a cluster randomized controlled study design with baseline measurements in Spring 2010 (5-6th grade) and follow-up to be collected in Spring 2012 (7-8th grade) (Figure 1).

Estimation of sample size - power calculations

Sample size calculations were performed prior to the study. Conventional levels of statistical power (0.8) and level of significance (0.05) were used in the two-sided test The minimum detectable effect size between groups was determined at 60 counts/min, representing an approximate 10% difference in the outcome measure at follow-up. We used data from the Danish part of the European Youth Heart Study (EYHS)[29] to estimate the variability in the change in physical activity from baseline to follow-up. Data from the EYHS was also used to estimate the between school variation in physical activity in order to control for clustering within schools (ICC = 0.011). The between school variation was estimated while controlling for most of the school matching variables described below. All analyses were performed with statistical software STATA v10 using the modules Sampsi and Sampclus. The calculations showed that a minimum of 12 schools (6 control and 6 intervention schools) were required, based on an average number of 100 students per school.

Recruitment and randomisation

Denmark is divided into five regions with approximately 20 municipalities in each region. The municipalities are responsible for health promotion and school education at the primary level (kindergarten to 10th grade). The main responsibility of the regions is to provide health care.

In spring 2009, all municipalities in the Region of Southern Denmark were invited to participate in the SPACE study. Five municipalities (Esbjerg, Nordfyn, Varde, Vejle and Sønderborg) out of 22 accepted the invitation and were asked to enroll public schools that contained 8th grade. A total of 28 schools were recommended or deemed eligible by the municipalities, however, schools were considered ineligible for the study based on the following criteria: a) if placed in the countryside with more than 25% of all students living further than 2 km crow fly distance from the school; and b) if the majority of all students were non native Danish. Five schools were excluded by the research team because they did not meet the above mentioned criteria. This resulted in an enrollment of

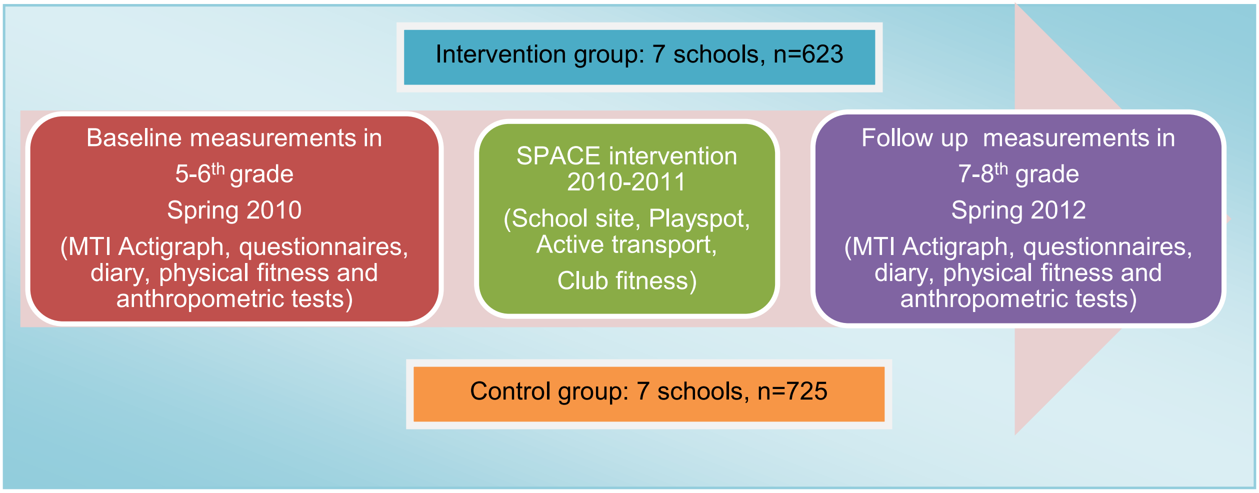

Figure 1 Illustration of the SPACE study design.

23 schools in the five municipalities. To reduce the number of schools required according to the sample size estimation, we developed and used an audit tool. The audit tool consisted of a total of 8 school characteristics consisting of 4 objective and 4 subjective qualities from the 23 schools. The objective characteristics were: 1) crow-fly distance from residence to school for 5-6th graders, 2) area household income, 3) area education level, and 4) area ethnicity distribution. The information was obtained from Statistics Denmark and with the use of Geographic Information System (GIS). The subjective information, which was based on interviews with municipality consultants and managing school personnel at each school site, consisted of the following parameters: 1) school district urbanity, 2) condition and characteristics of school outdoor areas, 3) school health policy, and 4) active transport in the local area. After visits at the school sites, 2 schools declined to participate in the project resulting in 21 eligible schools. A Spearman rank correlation analysis was conducted with the standardized values of the eight measures between the 21 schools. As part of the selection process, it was a given condition that Esbjerg and Varde municipalities participated in the project with four schools each, and the municipalities of Nordfyn, Vejle and Sønderborg participated with two schools each. The seven best matched pairs were selected for randomization, resulting in a total of 14 schools participating in the study. The schools were then randomized in an intervention group and a control group. Based on the eight school characteristics and the limited number of schools, a matched pair design was applied to optimize the randomization [30]. In order to increase power however an unmatched analysis of the matched data was adopted [30]. A flowchart illustrating the recruitment and randomisation can be seen in Figure 2.

Parents of the participating children received a passive informed consent form that explained the nature and procedures of the study. If parents and/or their child(ren) did not want to participate, they could withdraw during any stage. The study has been reviewed by the Danish Ethical Committee and they concluded that formal ethics approval was not required. The study is registered and listed in the Danish Data Protection Agency (reference number: 2009-41-3628).

Intervention components

The multicomponent intervention comprises four main areas: 1) the school's outdoor areas, 2) playspots 3) active transport and 4) club fitness. A total package of 11 intervention components combining both organizational and physical environment changes will be implemented in all 7 intervention schools. An overview of the 11 intervention components can be seen in Figure 3. A detailed description of the 11 intervention components can be seen at: http://www.forebyggelsescenter.dk.

Implementation, organizing and anchoring

The development of the study design and the implementation of the intervention are carried out in close partnerships between the research team at University of Southern Denmark, the Region of Southern Denmark, the five municipalities, and the 14 schools. The Region of Southern Denmark serves as coordinator in relation to the municipalities and participates with process management. One fulltime employee is allocated by the region for this task. The municipalities involved are expected to finance intervention costs with support from various funds and a halftime employee is available for the development and coordination of the local interventions. The schools, which

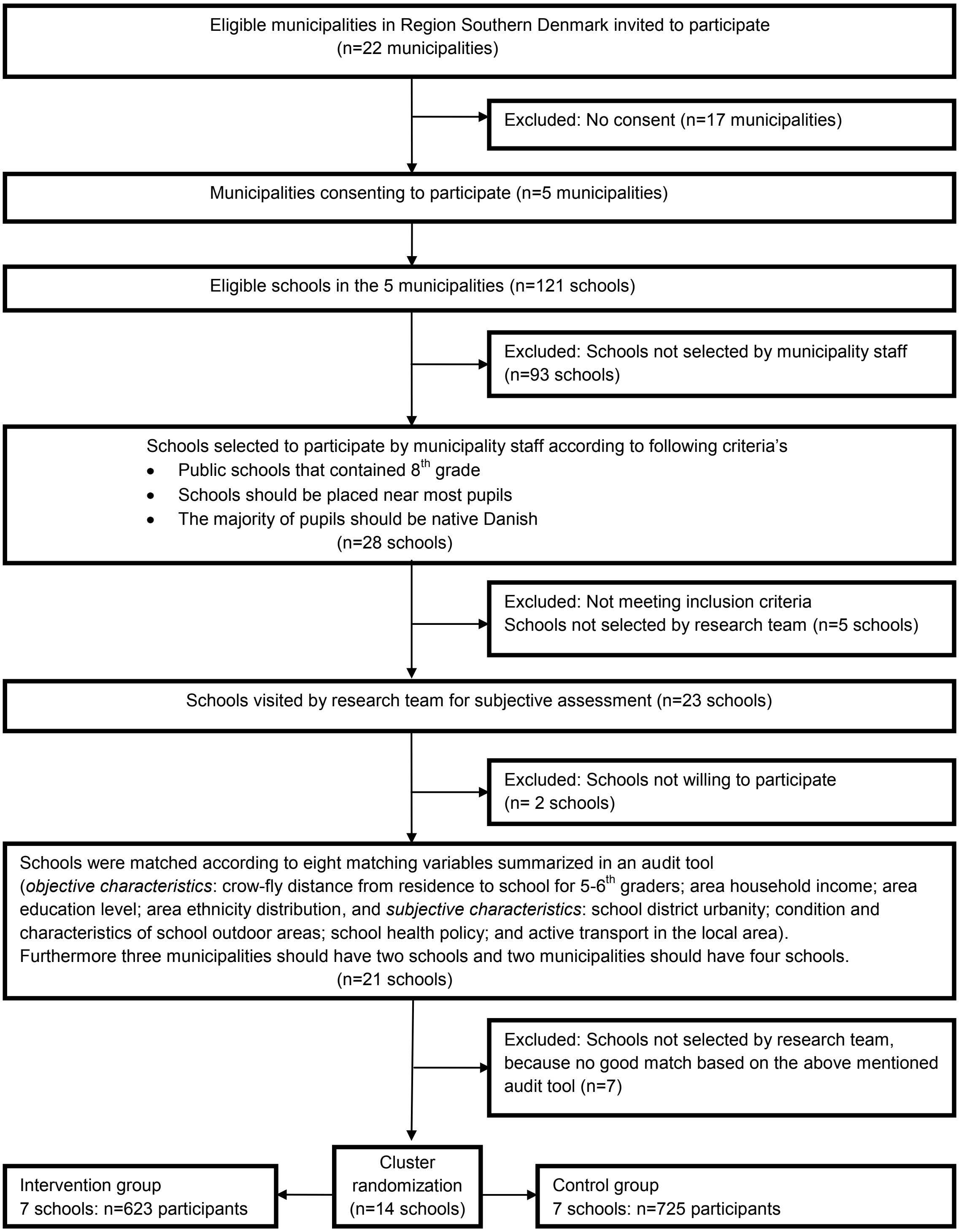

Figure 2 Flow diagram of recruitment and randomization of schools in the SPACE for physical activity study.

are the targets of the interventions, performed the tasks of specifying, adapting and implementing the interventions. Thus, a primary aspect of the intervention was to involve school teachers, local institutions and organizations in anchoring the initiatives. It is however, important to establish that the overall content of the 11 intervention components were defined prior to the project start, and are the same in all 7 intervention schools. In Denmark the conditions for collaboration between universities and municipalities are present, since the municipalities have

Intervention component	Description	Type of intervention
School Outdoor area	The objective is to enhance the schoolyard as an arena for self-organized play, sports and PA – both during and outside school hours. The school outdoor area will be redesigned or remodelled based on student input to facilities and environments promoting PA. On average each school has 12.000€ to upgrade the part of the school yard intended for lower secondary education.	Physical environment
Playspot	A new venue for physical activities and socializing for adolescents. Normally playgrounds are intended for small children. However, Norwegian and Danish examples show that it is possible to create playgrounds that appeal to youth and adults. This part of the intervention will be undertaken in dialog with the Danish Foundation for Culture and Sports Facilities and an Architect & Landscape company. The playspots will be established by Summer 2011, and the construction cost of each playspot is on average 151.000€ (65.000-250.000€)[44].	Physical environment
Active transport/ Safe routes to school	The objective is to encourage more children to cycle and walk in the local area. To increase active transportation the effort comprises: analyzing the possibilities for and limitations to cycling and walking, together with mapping of accident locations; prioritizing traffic regulation: speed-reducing mechanisms, car-free zones, traffic-sign warnings, accident-reducing facilities at intersections, one-way traffic, school traffic patrols, etc.; marking and signposting safe routes for walking and cycling. To finance this part we applied for funding from the Danish Road Council in fall 2010.	Organizational & Physical environment
Club fitness	The objective is to provide possibilities for students to participate in organized teen-fitness activities irrespective of the individual user's physical capacity, with flexible opening hours at a low cost. In studies of barriers to PA it has been demonstrated that adolescents, despite a lack of interest, are attracted to fitness, dance, aerobics, and workout [45]. This effort will be jointly undertaken with a special unit under the Danish Sports Associations DGI/DIF which support local initiatives for non-profit fitness (www.foreningsfitness.dk). The effort comprises the following: the establishment of non-profit organized fitness in each of the intervention districts; professional support from sport organizations to local organizations; and professional exercise instruction to provide guidance and support for any newcomers, based on the experience of similar exercise instruction elsewhere.	Organizational & Physical environment
Physical Activity Policy	Every school and their school board have prepared and adopted a physical activity policy with aims, goals, and objectives for how to promote PA in the school domain, including the school outdoor areas, outdoor breaks, active transportation, and school projects about physical activity.	Organizational
Kick starters	A kick starter is a specially trained teacher that initiates and motivates for PA during breaks, especially for students in 6-8th grades. 3-4 teachers from each intervention school have by January 2011 completed the "kick starter education" offered by University College Lillebaelt in the Region of Southern Denmark, and the intervention was implemented subsequently.	Organizational
School play patrol	Students in 6-7th grade are educated in initiating play and games for minors. From each school 8-10 students were trained for the school play patrol. Besides the promotion of PA and play, the purpose of the play patrol is to improve the social environment among students at school across age, gender and social class. This effort was developed and implemented jointly with the Danish Cancer Society that for several years has been working on the development of play and movement in school yards (www.legepatrulje.dk). The students completed the school play patrol education in fall 2010, and the intervention was implemented subsequently.	Organizational
Outdoor recess and open access to gym or sports hall	Mandatory outdoor recess all year for students in 6-9th grade was implemented in the intervention schools by August 2010. The students will be outdoors in the school breaks, for at least 20 minutes every day. Some schools have the possibility of open access to the school gym or sports hall in recess during winter, which can substitute the outdoor break.	Organizational
School traffic patrol	Students from 6th grade and above have the opportunity to attend the school traffic patrol. The main purpose is to help minors cross the streets near the school. The programme is offered by the Danish Road Safety Council (www.sikkertrafik.dk), and the local police department educate the student. In fall 2010, selected students received the training, and the school traffic patrol was implemented subsequently.	Organizational
Cyclist education	All students in 6-7th grade are educated in good and safe cyclist behaviour. The cyclist safety programme is offered by the Danish Road Safety Council (www.sikkertrafik.dk). After training courses, the students will complete a cycle safety test containing both practical and theoretical elements.	Organizational
School projects	In a scheduled week (week 42 in October) in 2010 and 2011 there will be a week focused on learning about and doing PA during school lessons. The purpose is to increase the students' knowledge about PA and health, and to be physically active for at least 60 minutes each day during the school day. At the end of the week, on Friday, the National School Exercise Day takes place, and in 2010 the national information campaign Get Moving (Getmoving.dk) took place in same week with the aim of spreading the message that children and young people must be active for at least 60 minutes a day.	Organizational

Figure 3 Intervention components in the SPACE for physical activity study.

taken on the responsibility for a number of health promotion and disease prevention tasks following a comprehensive structural reform in 2007. Thus, the original initiative to develop the project originates from collaboration between Region of South Denmark and municipalities in the region. Furthermore, the proposed collaborative structure is already familiar to the partners.

Data collection procedures

The evaluation of the SPACE study is split into three main parts: 1) quantitative evaluation of physical activity, physical fitness, and determinants for physical activity, 2) anthropologic evaluation of the intervention effect in two schools, and 3) process and health economic evaluations of the intervention. This study protocol focuses on the first part that consists of objective assessment of PA by accelerometry measures, physical fitness and anthropometric tests, questionnaires and activity diaries (table 1). Below is a thorough description of the quantitative methods of measurements used in the study.

Pilot study

Prior to baseline (in February 2010), a comprehensive pilot study was conducted containing objective measurements of PA, questionnaires, diaries, and physical fitness and anthropometric tests. Focus group interviews with adolescents in 5-6th grade and interviews with the school personnel at the pilot school were carried out to improve data collection procedures. Based on the experiences from the pilot study, smaller changes and adjustments were made.

Baseline

The baseline measurements took place in April-June 2010. Data collection was conducted simultaneously at the intervention school and the paired control school. Three to four weeks before data collection, information materials were sent to the schools describing details, collection measures, and detailed timetables of the study. At every school, an employee was appointed as a contact person being responsible for: a) distributing of study materials to the students and involved teachers, b) collecting all diaries and accelerometers, c) booking of the school's computer lab for the questionnaire session, and d) reserving the school gym for physical fitness and anthropometric tests.

Questionnaires

Three different questionnaires were used - for students, parents, and school management, respectively. All questionnaires were internet based.

Student Questionnaires The questionnaire for students contained a total of 48 questions on PA in leisure time,

Table 1 Description of baseline measurements April-June 2010, study population (adolescents: n = 1348, parents: n = 1348, school managements: n = 14), and response rate

Measurement	*Who*	*What*	*When*	*Response rate*
Questionnaire	Adolescents	Students filled out an e-survey in a school session with questions about physical activity, health, neighbourhood, school etc.	Duration: 30 minutes. During the week when wearing accelerometer in April-June 2010	97.4% (n = 1313)
Diaries	Adolescents	Every morning during one week the students filled out a diary with information about active transportation, and use of accelerometer	5 minutes each school day in 1 week (5 weekdays and 2 weekend days) simultaneously with wearing the accelerometer, in April-June 2010	96.6% (n = 1302)
Physical fitness and anthropometric tests	Adolescents	Students (without health problems) participated in a physical testing session of 11/2 hour including: body measurements (weight, height, waist circumference), running test, agility test, fitness test	Duration 11/2 hour, at the end of the week when wearing accelerometre. April-June 2010	93.9% (n = 1266)
MTI Actigraph	Adolescents	Students wore an accelerometer (MTI Actigraph, GT3X) in 7 days. 5 week days and 2 weekend days	All day in 1 week (5 weekdays and 2 weekend days) in April-June 2010	88.6%* (n = 1194)
Questionnaire	Parents	One parent (mother or father) completed an e-survey about parental physical activity and health behaviour, the neighbourhood etc.	Duration: 15 minutes. In April-June 2010	72.6% (n = 978)
Questionnaire	School management	A person from the school management completed an e-survey about the school social and physical environment, school politic etc.	Duration: 10 minutes. In April-June 2010	100% (n = 14)

*Response rate calculated as those wearing accelerometer for at least 9 hours in at least 4 days (time interval 7a m-11 pm. cut out blocks of non wear defined as 20 min of inactivity - consecutive zeroes).

sedentary behaviour, active transport, health related behaviour, self-reported health status, living conditions, school satisfaction, and assessment of neighbourhood (physical environment, perception of safety, social capital etc.) and possibilities for being physical active in the neighbourhood and school setting. The majority of questions have previously been validated and/or used in other (inter)national surveys about children and adolescents' health (e.g. Health Behaviour in School-aged Children)[31]. The students completed the questionnaire on a computer in one school lesson (45 minutes) during the week wearing accelerometers prior to the physical fitness and anthropometric tests. A teacher was present during the session and instructed the students how to get started and handed out a unique password to each of the students to access the questionnaire. The teacher helped if there were any questions or problems with comprehension. It took 30-40 minutes to complete the questionnaire. All students were instructed to fill out the questionnaire individually, without talking to classmates. In June 2010, participating schools were requested to conduct a session for (the few) students who were not present at the first questionnaire session. This increased the already high response rate from 94.3% to 97.4% (table 1).

Parent Questionnaires The questionnaire for parents contained questions on their own physical activity behaviours and their general overall health status as well as living conditions, assessment of neighbourhood (physical environment, perception of safety, social capital etc.) and the possibilities for being physically active in their neighbourhood. Only one parent for each student was requested to answer the questionnaire. Three to four weeks before the physical testing of students, parents received a unique password and a link to the questionnaire together with an information leaflet about the SPACE study. An estimated 10-15 minutes were projected to complete the questionnaire. In June 2010, a reminder was sent by the school intranet to those parents who did not initially fill out the questionnaire. This increased the response rate from 70.0% to 72.6% (table 1).

School Administrator Questionnaires The questionnaire for school management included questions about the school's physical, socio-cultural, educational and political environment, with a special focus on initiatives regarding PA and general health of the students. The questionnaire was completed by the school principal or a similar administrative contact person. The questionnaire was filled out during the data collection period in April-June 2010, and took approximately 10 minutes to complete.

Student Assessment Materials

MTI Actigraph Objective PA levels were obtained with the tri-axial Actigraph GT3X Activity Monitor[32]. The Actigraph detects acceleration in vertical, horizontal and transverse axes. All students who participated in the project were asked to wear an accelerometer for seven consecutive days on an elastic belt around the waist. Verbal and written instructions by trained professionals or by the research staff were given to the students regarding its

use. All children were instructed to wear the monitor continuously during the day. The data was recorded at 2 second epoch, with total PA to be expressed in counts per minute. The monitor has been previously validated in both children and adolescents against a range of different outcomes[33-35]. To increase participation, the adolescents or one of their parents were offered a free text message reminder on their phone every morning.

Active commuting Diary On weekdays when wearing the accelerometers, the students filled out a short diary during the first school lesson. The diary contained questions about transport to/from school and biking in general for the previous day. Furthermore at the end of the week, the students were asked about their use and reason for non-use of the accelerometer.

Physical fitness and anthropometric tests The test lasted approximately 11/2 hours for each class and was conducted in the school gym or a nearby sports hall during school hours. A test team consisted of 5-6 people; a test leader, and 4-5 trained assistants. The contact person and/or the class teacher were present during the physical fitness and anthropometric tests and helped where needed. At the beginning of the session, all adolescents were introduced to the program. All participants in the test received written feedback with their own results on the below mentioned measures and tests.

Anthropometry. Height, weight, and waist circumference were assessed by standard anthropometric procedures. Height was measured with a portable stadiometer (SECA Leicester portable Height Measure). Students were asked to take off their shoes and stand with their back to the stadiometer. The sliding head piece of the stadiometer was lowered so that the hair was pressed flat, and height was recorded to the nearest millimetre.

Body weight was measured with a medical scale (Tanita BWB-800S Digital Scale). The participants were asked to take off shoes and pants if wearing long pants, e.g. jeans. The weight was recorded to the nearest decigram.

Waist circumference was measured by use of body tape (Chasmors WM02 Body Tape, Chasmors Ltd., London, UK). The participants were asked to stand with their feet fairly close together with the weight equally distributed on each leg, and to breathe normally. The reading of the measurement was taken at the end of exhalation. The waist was measured at the navel level with the tape wrapped around the body in a horizontal alignment. The waist circumference was recorded to the nearest 0.5 cm.

Muscle strength was measured by hand grip with Takei TKK A5401 Digital Handgrip Dynamometer. The test was performed with maximal exertion using the dominant hand with a straight arm at the side of the body. The test was repeated twice while conducting the agility test in between. The higher of the two measurements was recorded.

Test of agility and speed. The Eurofit test is a 10 × 5 m shuttle run that measures running speed and agility (ability to move quickly and change directions while maintaining control and balance). This test requires the person to run back and forth between the two parallel lines 5 m apart five times (50 m in total) as fast as possible. Both feet must fully cross the line every time[36].

Aerobic fitness was measured with the Andersen running test. Two parallel lines 20 m apart were made on the floor. Before the test start all participants were equipped with a Polar heart rate monitor and were thoroughly informed about the procedures. Eight to twelve subjects participated in the test at the same time. Subjects ran from one line to the other. They had to touch the line on the floor with one hand, turn around and run back. After 15 seconds, the test leader signalled the end of the test using a whistle and the subjects stopped immediately and rested for the next 15 seconds. This procedure was followed for 10 minutes. Subjects ran as fast as they could in order to cover the longest possible distance during the 10 minutes test run, and this distance was the test result[37].

Isometric back muscle strength test was measured by researchers in clinical biomechanics. Although not directly related to the SPACE study, these measures were also taken during physical fitness testing to determine associations between back muscle strength and other relevant health outcomes e.g. physical activity levels.

Socio demographic and background variables were obtained from the questionnaires mentioned above, and from Statistics Denmark using the Danish Civil Registration System, which individually monitors information such as, gender, age, address, ethnicity, income and parental socioeconomic status for all residents of Denmark (each Danish citizen has a unique personal registration number).

Outcome measures

Primary, secondary, and predefined explorative outcome measures are registered at the Current Controlled Trials website[38]. An overview and description of the outcome measures is listed in Figure 4.

Statistical analysis

The effect of the intervention will be tested using multilevel mixed effects models adjusted for relevant potential confounding factors (e.g. baseline outcome measure, gender, age, family socioeconomic status and parents' PA level). Because of the cluster structure of the data, random effects for school will be included in all analyses. In Figure 4 the primary, secondary and predefined explorative outcome measures are listed, together with the type

Outcome measure	Description	Type of variable
Primary outcome measure(s)	1. Average daily physical activity measured with accelerometer (counts/min/child/day).	*Continuous*
	2. The primary outcome will be supported by analyses of time spent in moderate to vigorous activity and time spent sedentary measured with accelerometer (min/day).	*Continuous*
Secondary outcome measures	I. Overweight measured with waist circumference (cm)	*Continuous*
	II. Physical fitness measured with the Andersen-test (meter).	*Continuous*
	III. Active transport to/from school measured with diary (times/week).	*Ordered categorical*
	IV. Physical activity in recess periods measured with accelerometer (counts/min).	*Continuous*
Predefined explorative outcome measures	A. The primary and secondary outcome measures will be analyzed for four different subpopulations defined by: physical activity at baseline, parental socioeconomic status, weight status at baseline and gender.	*Continuous and ordered categorical, as described above*
	B. Grip strength measured with digital hand dynamometer (kg).	*Continuous*
	C. Time spent on television viewing, computer or other sedentary activities measured with questionnaire (hours/day).	*Ordered categorical*
	D. Time spent on biking measured with diary (times/week)	*Ordered categorical*
	E. School satisfaction measured with questionnaire (index five categories of liking school)	*Ordered categorical*
	F. Self-rated health measured with questionnaire (index four categories)	*Ordered categorical*
	G. Social network measured with questionnaire (time spent with friends and family)	*Ordered categorical*
	H. Experience of subjective health complaints measured with questionnaire (numbers and times of multiple experienced health complaints)	*Ordered categorical*

Figure 4 Primary, secondary, and predefined explorative outcome measures.

of variable in each outcome measure. All primary and secondary outcome measures are continuous except for the secondary outcome measure no III regarding active transport to/from school, which is an ordered categorical variable that measures times of active transport during a week. The primary and secondary outcome measures are primarily based on assumptions of a normal distribution; however the secondary outcome measure about active

transport is based on the assumption of a Poisson distribution. When more than one test is used, it is always important to be aware of the risk of type 1 error inflation. We have selected a primary outcome measure, that is deemed the most important, and then we have estimated 4 secondary distinct outcome measures to be acceptable given the effect size and quality of the study. Because of the risk of type 1 error, the results from the secondary outcome measures will be treated with caution.

The statistical software package, STATA (v.11.0, Texas, USA) will be used. The effectiveness of the intervention on adolescent physical activity will be assessed utilising an intention to treat approach[39].

Discussion

A total of 1348 adolescents in 5-6th grade in 56 classes in 14 schools in the Region of Southern Denmark participated in the baseline study (623 in intervention schools and 725 in control schools). In general, the response rate among students was high for all baseline measurements obtained from the questionnaire (97.4%), diaries (96.6%), physical fitness and anthropometric tests (93.9%), MTI Actigraph (min. 9 hr in 4d) (88.6%). A total of 978 parents responded to the parental survey (response rate: 72.6%), while all 14 school administrators filled out corresponding questionnaires (table 1). Table 2 presents baseline characteristics of the study population. Overall, 48.4% were girls, average age 12.5 years, BMI 18.9 kg/m^2, height and weight 47.2 kg and 157.6 cm, waist circumference 69.8 cm, average meters covered in the fitness running test were 1000.3 meters, and average physical activity counts per minute measured by accelerometer were 609.3 counts/min. There were no significant differences between intervention (n = 623) and control (n = 725) groups at baseline according to above mentioned characteristics.

The purpose of this article was to give an overview of the study design in the SPACE Study as there is a growing demand for describing complex behaviour change interventions and to produce greater clarity about the functional components of those interventions[40]. In a review by Michie and colleagues, it was concluded that interventions were described in detail in only 5-30% of experimental studies[41].

Strengths of the SPACE study are the cluster randomized controlled study design and that it builds on a social ecological approach and thus acknowledges the importance of comprehensive multilevel interventions. Furthermore the study is conducted in a real life setting, where the usual staffs in municipalities and schools, under expert guidance from the research group, carries out the implementation of the intervention components. This also means that any findings of the trial are more generalisable because they are performed in the setting in which they are most likely to be implemented. This complies with the needs for more effectiveness trials, defined as interventions delivered under real-world conditions [42,43].

On the other hand, it is very important and a great challenge to ensure a certain degree of conformity of the implementation process when conducting effectiveness trials. We try to account for this issue in the study design, where process- and health economic analyses together with anthropologic observations will be carried out to address this issue concurrently with the effectiveness analysis. One relevant aspect with regards to this was that the financial crisis developed prior to the intervention, challenged fundraising for the built interventions considerably. Any financial difference will be examined in the future after the study is over. The implementation process is carefully followed to document what actually happens, and the municipal coordinators are responsible for conducting ongoing feedback and fill out evaluation forms of how the implementation process is carried out in the intervention schools, and to monitor if any extraordinary efforts in relation to physical activity and health are about to take place in the control schools.

We obtained a high response rate in the baseline measurements. Especially the measurements among adolescents conducted during the school day (questionnaire, diary, physical fitness and anthropometric tests) revealed a very high response rate (>93%). This demonstrates one of the strengths of conducting school based intervention research because the students had to be present in the school. Moreover an important explanation for the high response rate was the passive informed consent form used in the study. The school management and the contact person also played important roles by communicating information about the project and motivating students, parents, and other involved teachers to participate in the project. Even small differences between schools regarding engagement and commitment to the project are important as this can result in possible biases.

Physical activity is notoriously difficult to measure, and there are limitations of accelerometry as well as limitations in self-reported measurements of PA. We believe that the triangulation of methods used in this study will try to overcome measurement difficulties. A good example is that in Denmark, bicycling is an important mode of transportation but poorly registered by the accelerometer. Thus, measurement of cycling via self-reported measurements were applied. In our study, 78.4% of students in 5-6th grade always or most of the time cycle to school according to the student questionnaire.

Finally it is worth to mention that we found no significant differences between the intervention and control group at baseline, suggesting that the matching with the audit tool was appropriate for creating equal groups.

Table 2 Baseline participant characteristics

	Total Baseline population (n = 1194-1348)*	Intervention group (n = 574-623)*	Control group (n = 633-725)*	Difference between intervention and control (p-value)
Gender, No. (%)				
Girls	652 (48.4)	307(49.3)	345 (47.4)	38
Boys	696 (51.6)	316 (50.7)	380 (52.6)	64 (p = .54)
Age, mean (SD), y	12.5 (0.63)	12.4 (0.63)	12.5 (0.62)	0.05 (p = .17)
BMI, mean (SD), kg/m²	18.9 (3.01)	18.9 (3.00)	18.8 (3.03)	0.09 (p = .59)
Weight, mean (SD), kg	47.2 (10.18)	47.2 (10.04)	47.2 (10.31)	0.08 (p = .89)
Height, mean (SD), cm	157.6 (8.37)	157.5 (8.20)	157.7 (8.53)	0.22 (p = .65)
Waist circumference, mean (SD), cm	69.8 (8.77)	70.1 (8.83)	69.5 (8.71)	0.67 (p = .17)
Physical fitness, mean (SD), meters	1000.3 (103.47)	1000.6 (103.09)	1000.1 (103.89)	0.52 (p = .93)
Physical activity, mean (SD), counts/min#	609.3 (241.4)	596.7 (229.6)	620.3 (250.7)	19.4 (p = .09)

* n varies due to different sources of data. #Mean counts/min calculated using only the vertical axis.

Conclusion

This manuscript provides a description of the study design, data collection, intervention components and the implementation of a multicomponent intervention study aiming at increasing physical activity levels of adolescents. The SPACE study is based on a social ecological framework, which is designed to implement organizational and structural changes in the school environment. In conclusion the SPACE Study is believed to contribute to new insights regarding multicomponent physical activity interventions in youth, and the results of the study may be used a basis for the development of further health enhancing PA planning in school aged adolescents.

Acknowledgements
The SPACE Study is part of The TrygFoundation Centre for Applied Research in Health Promotion and Disease Prevention. We thank the participating schools, their teachers and students, field staff who assisted in data collection, and others involved in the project. Thanks to Pernille Due and Chalida Svastisalee, National Institute of Public Health, University of Southern Denmark for useful comments and proof-reading.

Authors' contributions
JT is the Principal Investigator of the SPACE study. MT led the writing for this manuscript with contributions and critical comments from the other three authors. MT and LBC were responsible for and carried out the quantitative data collection procedures in the SPACE study. All authors contributed to the research design, provided comments on the drafts, and read and approved the final manuscript.

Competing interests
The authors declare that they have no competing interests.

Received: 23 June 2011 Accepted: 10 October 2011
Published: 10 October 2011

References

1. Floriani V, Kennedy C: **Promotion of physical activity in children.** *Curr Opin Pediatr* 2008, **20**:90-95.
2. Strong WB, Malina RM, Blimkie CJR, Daniels SR, Dishman RK, Gutin B, *et al*: **Evidence based physical activity for school-age youth.** *Journal of Pediatrics* 2005, **146**:732-737.
3. Andersen LB, Sardinha LB, Froberg K, Riddoch CJ, Page AS, Anderssen SA: **Fitness, fatness and clustering of cardiovascular risk factors in children from Denmark, Estonia and Portugal: the European Youth Heart Study.** *Int J Pediatr Obes* 2008, **3(Suppl 1)**:58-66.
4. Janssen I, LeBlanc AG: **Systematic review of the health benefits of physical activity and fitness in school-aged children and youth.** *International Journal of Behavioral Nutrition and Physical Activity* 2010, **7.**
5. Andersen LB, Hasselstrom H, Gronfeldt V, Hansen SE, Karsten F: **The relationship between physical fitness and clustered risk, and tracking of clustered risk from adolescence to young adulthood: eight years follow-up in the Danish Youth and Sport Study.** *Int J Behav Nutr Phys Act* 2004, **1**:6.
6. Andersen LB, Harro M, Sardinha LB, Froberg K, Ekelund U, Brage Sr, *et al*: **Physical activity and clustered cardiovascular risk in children: a cross-sectional study (The European Youth Heart Study).** *The Lancet* 2006, **368**:299-304.
7. Telama R, Yang X, Viikari J, Valimaki I, Wanne O, Raitakari O: **Physical activity from childhood to adulthood: a 21-year tracking study.** *Am J Prev Med* 2005, **28**:267-273.
8. Sundhedsstyrelsen: **Undersøgelse af 11-15 åriges livsstil og sundhedsvaner 1997 - 2008.** 2010, Ref Type: Report.
9. Salmon J, Timperio A: **Prevalence, trends and environmental influences on child and youth physical activity.** *Med Sport Sci* 2007, **50**:183-199.
10. van Sluijs EM, McMinn AM, Griffin SJ: **Effectiveness of interventions to promote physical activity in children and adolescents: systematic review of controlled trials.** *BMJ* 2007, **335**:703.
11. Dobbins M, De Corby K, Robeson P, Husson H, Tirilis D: **School-based physical activity programs for promoting physical activity and fitness in children and adolescents aged 6-18.** *Cochrane Database of Systematic Reviews* 2009.
12. Nichol ME, Pickett W, Janssen I: **Associations between school recreational environments and physical activity.** *J Sch Health* 2009, **79**:247-254.
13. Lanningham-Foster L, Foster RC, McCrady SK, Manohar CU, Jensen TB, Mitre NG, *et al*: **Changing the school environment to increase physical activity in children.** *Obesity* 2008, **16**:1849-1853.

14. World Health Organization: **What is the evidence on school health promotion in improving health or preventing disease and, specifically, what is the effectiveness of the health promoting schools approach?** 2006, Ref Type: Report.
15. Naylor PJ, McKay HA: **Prevention in the first place: schools a setting for action on physical inactivity.** *British Journal of Sports Medicine* 2009, **43**.
16. Stone EJ, McKenzie TL, Welk GJ, Booth ML: **Effects of physical activity interventions in youth - Review and synthesis.** *American Journal of Preventive Medicine* 1998, **15**:298-315.
17. King AC: **Theoretical Approaches to the Promotion of Physical Activity. Forging a Transdisciplinary Paradigm.** *American Journal of Preventive Medicine* 2002, **23**.
18. Pate RR, Davis MG, Robinson TN, Stone EJ, McKenzie TL, Young JC: **Promoting physical activity in children and youth: a leadership role for schools: a scientific statement from the American Heart Association Council on Nutrition, Physical Activity, and Metabolism (Physical Activity Committee) in collaboration with the Councils on Cardiovascular Disease in the Young and Cardiovascular Nursing.** *Circulation* 2006, **114**.
19. Pate RR, Saunders R, Dishman RK, Addy C, Dowda M, Ward DS: **Long-term effects of a physical activity intervention in high school girls.** *Am J Prev Med* 2007, **33**:276-280.
20. Giles-Corti B, Timperio A, Bull F, Pikora T: **Understanding Physical Activity Environmental Correlates: Increased Specificity for Ecological Models.** *Exercise and Sports Science and Reviews* 2005, **33**:175-181.
21. Sallis JF, Cervero RB, Ascher W, Henderson KA, Kraft MK, Kerr J: **An ecological approach to creating active living communities.** *Annu Rev Public Health* 2006, **27**:297-322.
22. Stokols D: **Establishing and Maintaining Healthy Environments. Toward a Social Ecology of Health Promotion.** *American Psychologist* 1992, **47**:6-22.
23. Sallis FJames, Owen Neville, Fisher BEdwin: **Ecological models of health behavior.** In *Health Behavior and Health Education. Theory, Research, and Practice.* Edited by: Karen Glanz, Barbara K Rimer, Viswanath K. San Francisco: Jossey-Bass; 2008:465-485.
24. Pilgaard M: **Danskernes motions- og sportsvaner 2007 - nøgletal og tendenser.** Idrættens Analyseinstitut; 2008, Ref Type: Report.
25. Armstrong N, Welsman JR: **The physical activity patterns of European youth with reference to methods of assessment.** *Sports Medicine* 2006, **36**:1067-1086.
26. Sallis JF, Prochaska JJ, Taylor WC: **A review of correlates of physical activity of children and adolescents.** *Med Sci Sports Exerc* 2000, **32**:963-975.
27. Riddoch CJ, Andersen LB, Wedderkopp N, Harro M, Klasson-Heggebo L, Sardinha LB, *et al*: **Physical activity levels and patterns of 9-and 15-yr-old European children.** *Medicine and Science in Sports and Exercise* 2004, **36**:86-92.
28. Campbell MK, Elbourne DR, Altman DG: **The CONSORT statement for cluster randomised trials.** *Medicina Clinica* 2005, **125**:28-31.
29. Riddoch C, Edwards D, Page A, Froberg K, Andersen SA, Wedderkopp N, *et al*: **The European Youth Heart Study - Cardiovascular Disease Risk Factors in Children: Rationale, Aims, Study Design, and Validation of Methods.** *Journal of Physical Activity and Health* 2005, **2**:115-129.
30. Diehr Paula, Martin CDonald, Koepsell Thomas, Cheadle Allen: **Breaking the matches in a paired t-Test for community interventions when the number of pairs is small.** *Statistics in Medicine* 1995, **14**:1491-1504.
31. **Health Behaviour in School aged children.** 2011 [http://www.hbsc.org], Ref Type: Internet Communication.
32. **Actigraph.** 2011 [http://www.theactigraph.com/products/gt3x/], Ref Type: Internet Communication.
33. Brage S, Franks P, Ekelund U, Wareham N: **Reliability and validity of the ActiHeart: A single-piece instrument that measures acceleration, heart rate, and heart rate variability.** *Obesity Research* 2003, **11**:A100.
34. Brage S, Wedderkopp N, Franks PW, Andersen LB, Froberg K: **Reexamination of validity and reliability of the CSA monitor in walking and running.** *Medicine and Science in Sports and Exercise* 2003, **35**:1447-1454.
35. Ekelund U, Sjostrom M, Yngve A, Poortvliet E, Nilsson A, Froberg K, *et al*: **Physical activity assessed by activity monitor and doubly labeled water in children.** *Medicine and Science in Sports and Exercise* 2001, **33**:275-281.
36. Eurofit: *Eurofit Tests of Physical Fitness.* 2 edition. Strasbourg; 1993.
37. Andersen LB, Andersen TE, Andersen E, Anderssen SA: **An intermittent running test to estimate maximal oxygen uptake: the Andersen test.** *Journal of Sports Medicine and Physical Fitness* 2008, **48**:434-437.
38. **International Standard Randomised Controlled Trial Number Registration.** 2011 [http://www.controlled-trials.com/isrctn/], Ref Type: Internet Communication.
39. White IR, Horten NJ, Carpenter J, Pocock SJ: **Strategy for intention to treat analysis in randomised trials with missing outcome data.** *BMJ* 2011, **342**:910-912.
40. Godlee F: **Publishing study protocols: making them visible will improve registration, reporting and recruitment.** *BMC News and views* 2001, **2**.
41. Michie S, Fixsen D, Grimshaw JM, Eccles MP: **Specifying and reporting complex behaviour change interventions: the need for a scientific method.** *Implementation Science* 2009, **4**.
42. Glasgow RE, Lichtenstein E, Marcus AC: **Why don't we see more translation of health promotion research to practice? Rethinking the efficacy-to-effectiveness transition.** *Am J Public Health* 2003, **93**:1261-1267.
43. Bartholomew LK, Parcel GS, Kok G, Gottlieb NH: *Intervention Mapping, Designing theory-and Evidence-based Health Promotion* New York: McGraw-Hill Higher Education; 2001.
44. **Peter Holst Arkitektur og Landskab. Playspots.** *Copenhagen, The Danish Foundation for Culture and Sports Facilities* [Lokale og Anlægsfonden]; 2011, Ref Type: Catalog.
45. Rees R, Kavanagh J, Harden A, Shepherd J, Brunton G, Oliver S, *et al*: **Young people and physical activity: a systematic review matching their views to effective interventions.** *Health Educ Res* 2006, **21**:806-825.

Pre-publication history

The pre-publication history for this paper can be accessed here:
http://www.biomedcentral.com/1471-2458/11/777/prepub

doi:10.1186/1471-2458-11-777
Cite this article as: Toftager *et al.*: SPACE for physical activity - a multicomponent intervention study: study design and baseline findings from a cluster randomized controlled trial. *BMC Public Health* 2011 **11**:777.

Submit your next manuscript to BioMed Central and take full advantage of:

- **Convenient online submission**
- **Thorough peer review**
- **No space constraints or color figure charges**
- **Immediate publication on acceptance**
- **Inclusion in PubMed, CAS, Scopus and Google Scholar**
- **Research which is freely available for redistribution**

Submit your manuscript at
www.biomedcentral.com/submit

BioMed Central
